# Retraction: “The Role of the Extracellular Matrix Protein Mindin in Airway Response to Environmental Airways Injury”

**DOI:** 10.1289/ehp.1003339RET

**Published:** 2016-04-01

**Authors:** Sarah Frush, Zhuowei Li, Erin N. Potts, Wanglei Du, Jerry P. Eu, Stavros Garantziotis, You-Wen He, W. Michael Foster, John W. Hollingsworth

Environ Health Perspect 119(10):1403–1408 (2011), doi: 10.1289/ehp.1003339

This article is being retracted at the request of the authors because of concerns about the accuracy of the initial data from the animal physiology laboratory at Duke University. The authors re-exported source data from the animal ventilator (FlexiVent) and compared the output with the raw data originally received from the animal pulmonary physiology laboratory. The results from these initial comparisons suggested potential inconsistencies in the data, so the authors requested that an independent laboratory replicate the experiments of animal airway physiology presented in Figure 1 and Figure 4. The results of the replicated experiments validated the originally reported central role of mindin in airway hyper-responsiveness after exposure to either lipopolysaccharide (LPS) or ozone.

Because the animal physiology laboratory at Duke University also analyzed cytokines, the authors had an independent laboratory replicate experiments to analyze the original role of cytokines reported in Figure 3 and Figure 6. In these replicate studies, which were limited in the number of animals and samples tested, the authors did not observe the same results and could not definitively determine whether or not the findings were valid.

The inconsistent results led the authors to take a conservative approach, and they agreed to retract this paper. They regret any inconvenience to the scientific community.

